# Describing a National Chatbot Deployed by the Ministry of Health in Malawi During the COVID-19 Pandemic: Retrospective Data Analysis

**DOI:** 10.2196/80960

**Published:** 2026-07-16

**Authors:** Isaach Ndemera, Yu-Ting Sunny Hsieh, Kingston Chirwa, Selemani Ngwira, Alanna Denny, Tsung Shu Joseph Wu, Hsin-yi Lee, Griphin Baxter Chirambo, Simeon Luka Yosefe, Ben Chilima, Matthew Kagoli, Balwani Mbakaya, Kwong Leung Joseph Yu, John O'Donoghue

**Affiliations:** 1 Research Department Luke International Mzuzu Malawi; 2 Graduate Institute of Environmental Engineering National Taiwan University Taipei Taiwan; 3 Luke International Mzuzu Malawi; 4 Malawi eHealth Research Centre Mzuzu University Mzuzu Malawi; 5 School of Public Health Department of Medicine & Health University College Cork Cork Ireland; 6 Department of Overseas Mission Pingtung Christian Hospital Pingtung Taiwan; 7 Nursing and Midwifery Department, Faculty of Health Sciences Mzuzu University Mzuzu Malawi; 8 National Statistics Office Lilongwe Malawi; 9 Public Health Institute of Malawi Lilongwe Malawi; 10 Department of Public Health University of Livingstonia Mzuzu Malawi; 11 School of Medicine University College Dublin Dublin, Leinster Ireland; 12 Application of Science to Simulation-based Education and Research on Training (ASSERT) Research Centre University College Cork Cork Ireland; 13 Business Information Systems University College Cork Cork Ireland

**Keywords:** Africa, COVID-19, pandemic, chatbot, social media, digital health, preparedness, response, risk communication

## Abstract

**Background:**

Malawi was a pioneer among African countries in implementing a coordinated, government-led effort to streamline COVID-19 support using digital health tools. In response to the pandemic, a COVID-19 WhatsApp chatbot was developed to support the public with information, symptom reporting, and service navigation during the pandemic.

**Objective:**

This study describes the national deployment, functionality, and use patterns of the WhatsApp chatbot during the COVID-19 pandemic in Malawi.

**Methods:**

A retrospective descriptive analysis of chatbot interaction data from May 2020 to May 2023 was conducted. User engagement with key chatbot functions was summarized using descriptive statistics, and time-series analysis was used to compare trends in reported COVID-19 cases with access patterns to the chatbot, the emergency operation call center, and Chipatala cha pa Foni (a national hotline initiative).

**Results:**

The chatbot was accessed 347,117 times, with 70.8% (n=245,895) of validated WhatsApp accesses focused on COVID-19 statistics. Chatbot use patterns showed temporal alignment with COVID-19 case trends, particularly during the first and second pandemic waves and increases observed after lockdown events. Throughout the pandemic, chatbot downtime occurred in 38% (407/1070) of days, with the most prolonged period coinciding with the national vaccine rollout in 2021, during which vaccine-related functionalities were introduced. Following this expansion, the chatbot recorded 198 COVID-19 vaccine–related rumors and 644 accesses to vaccine frequently asked questions. Compared with call-based services, the chatbot recorded higher overall interactions, whereas symptoms were more frequently reported through call center platforms.

**Conclusions:**

This study provides a descriptive account of the development and use of a national COVID-19 WhatsApp chatbot in Malawi. The findings highlight patterns of information-seeking behavior, variation in feature use, and the influence of system availability on engagement. These insights may inform the design, implementation, and sustainability of digital health communication tools in similar settings.

## Introduction

Digital tools hold significant potential to tackle the unique challenges posed by pandemics. Unlike other disasters, pandemics involve the spreading of pathogens through human contact, meaning that individual behavior plays a significant role in disease transmission. The fear of infection often leads to isolation and the concealment of symptoms to avoid stigma. Digital tools provide easy access to accurate information and support, helping reduce both the transmission of disease and the psychological strain associated with pandemics.

Among digital tools, social media platforms were widely used in health care during the pandemic [1,2]. The demand for timely and reliable information on disease symptoms and prevention surged during the COVID-19 pandemic, accompanied by a 10-fold increase in social media use [3]. In Africa, 216 million people (16.6% of the total population) used social media in 2019 [4]. Popular platforms such as Facebook, WhatsApp, and Facebook Messenger were widely adopted across the continent [4]. Integrating these platforms with chatbot features offers opportunities for information dissemination and public health surveillance, enabling participatory surveillance where individuals report symptoms directly [5].

Chatbots proved to be a critical component of the global response to COVID-19, with organizations including the World Health Organization (WHO) and Centers for Disease Control and Prevention actively promoting their use [6,7]. In May 2020, following the declaration of COVID-19 as a public health emergency, the WHO Regional Office for Europe and the United Nations Children’s Fund Europe and Central Asia Regional Office collaborated to rapidly develop HealthBuddy+, a chatbot tool [8]. Due to its initial success and increasing demand from the WHO and United Nations Children’s Fund country offices alongside national partners, HealthBuddy+ was expanded to support 16 languages, and a multifunctional mobile app was introduced. Despite limited resources, more than 15 African countries demonstrated remarkable digital adoption, in many cases surpassing high-income countries in leveraging chatbots for disease prevention, surveillance, and management [9-11]. Notable implementations include the WHO Health Alert chatbots in Egypt, Congo, and Zimbabwe [12]; the COVID-19 Telegram chatbot in Ghana to combat misinformation [13]; and the Mbaza chatbot in Rwanda providing COVID-19 information [14]. In South Africa and Senegal, WhatsApp chatbots were deployed to provide accurate COVID-19 information and facilitate testing [4].

Malawi, one of the pioneering countries in the region to implement a coordinated government-led effort to streamline digital health tools for COVID-19, offers a successful case study [15]. In response to the pandemic, the Ministry of Health (MOH) deployed a WhatsApp chatbot to support its COVID-19 response, engaging with approximately 10,000 citizens to self-register as COVID-19 suspected cases, report symptoms, and receive updates. Despite the rapid adoption of chatbots, their actual performance and contribution to national COVID-19 responses within the integrated digital health systems architecture remains largely unknown. Research on health chatbots in Africa is limited, with few empirical studies examining user experiences [11].

This study aimed to describe the national deployment, functionality, and use patterns of the WhatsApp chatbot during the COVID-19 pandemic in Malawi. The deployment of the Malawi WhatsApp chatbot offers a case study of how low- and middle-income countries can leverage existing digital platforms to support public health communication during emergencies. This analysis of quantitative interaction data characterizes the operational features of the chatbot and explores patterns of use that may inform the design and implementation of similar digital health tools in Africa.

## Methods

### WhatsApp Chatbot Design

The WhatsApp chatbot is linked to the MOH’s official WhatsApp number (+265-990-800-000), certified by Meta, allowing users seamless access. Once a user engages with the chatbot, the dialogue data are stored securely in an Amazon Web Services environment, which then feeds into a central PostgreSQL database (Figure S1 in Multimedia Appendix 1). This database is directly connected to Tableau (Salesforce, Inc) via MOH-built mediators to enable hourly updates for real-time data analysis and visualization. The output generated by the chatbot is based on a predefined algorithm that processes user inputs and delivers appropriate responses. The chatbot is designed to be flexible and adaptable, enabling the chatbot to evolve by incorporating new information or services as needed, thereby extending its utility and relevance to the users.

### Data Collection

This was a retrospective study of operational and service data collected through the MOH’s COVID-19 chatbot. The dialogue data recorded included the following:

- Bot ID: identifies the specific bot function (eg, COVID-19 statistics or second dose reminder)
- Date: the date of interaction (formatted as DD/MM/YYYY)
- Channel: the medium through which the chatbot was accessed (eg, web, application programming interface, or messaging)
- Metric: key performance indicators, such as enter_flow_count and msg_received_count
- Submetric: specific actions or functions within the chatbot (eg, “Nation statistics” or “Go to main menu”)
- Value: the count or frequency associated with each metric (eg, enter_flow_count)

Data were retrieved from the chatbot’s launch on May 29, 2020, extending throughout the pandemic until May 5, 2023, when the WHO declared an end to the COVID-19 global health emergency. This study focused on interactions with the COVID-19 WhatsApp chatbot, specifically using data from the application programming interface channel and analyzing enter_flow_count values across various submetrics. In addition to the COVID-19 WhatsApp chatbot data, secondary data sources were included, that is, the Public Health Emergency Operations Center COVID-19 dashboard and the emergency operation center (EOC) call center and Chipatala cha pa Foni (CCPF), a national hotline initiative, data were integrated to enhance the analysis.

### Data Analysis

**Figure 1:**
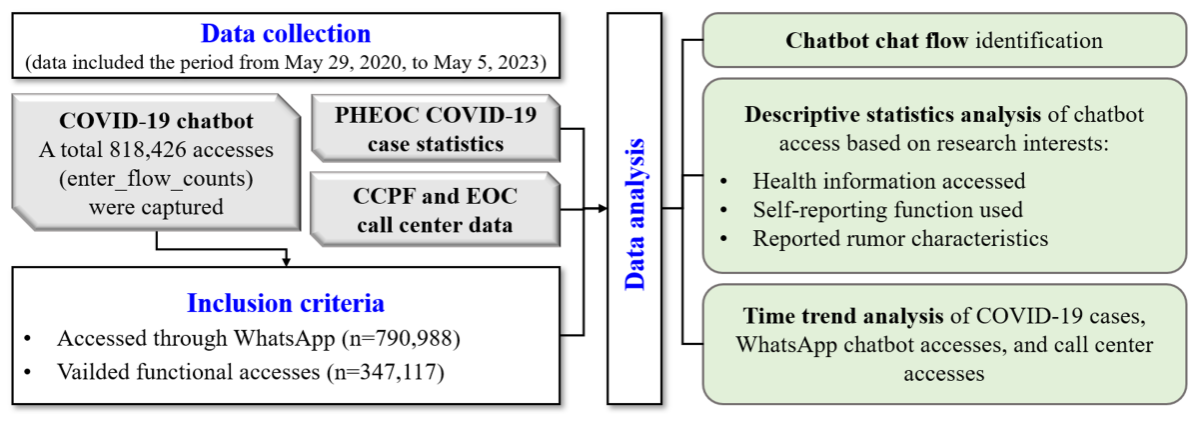
Retrospective study design flowchart. CCPF: Chipatala cha pa Foni; EOC: emergency operation center; PHEOC: Public Health Emergency Operations Centre.

To analyze access to the chatbot functions, descriptive statistics, including frequencies, percentages, and geographical information, were used. In this study, “access” was defined as a recorded entry into a chatbot function, represented by the enter_flow_count metric, and did not correspond to a unique user. The descriptive analysis was performed according to 3 categories: health information dissemination, self-reporting use, and rumor reporting characteristics. In addition, a time-series plot compared trends in COVID-19 cases with access patterns to the chatbot and the call center, providing insights into public interaction during the pandemic. A table of major COVID-19–related events was also compiled and incorporated with a timeline to support the interpretation of observed use patterns. Data analysis was performed using the R statistical software (version 4.3.3; R Foundation for Statistical Computing). The process began by identifying the chatbot’s chat flow to map the interaction design (Figure 1).

### Ethical Considerations

The Malawi National Committee on Research in the Social Sciences and Humanities reviewed and approved the study protocol, with approval number P.01/24/835. Due to the study’s retrospective nature and its use of preexisting data, obtaining individual consent retrospectively was not feasible. As a result, a written request was submitted to the MOH to gain access only to deidentified data. The deidentified data were protected and kept under lock and key according to the approved protocol (version 3.1). All key findings have been shared and discussed at a national workshop with participants from the MOH and the public.

## Results

The chatbot was officially launched on May 29, 2020, following the Malawi government’s declaration of a state of emergency due to the COVID-19 pandemic. The chatbot’s deployment was promoted through daily COVID-19 reports and official social media platforms such as the MOH Facebook page. Users could also share the WhatsApp chatbot with others using the share function. In total, 1% (3368/347,117) of the total relevant accesses involved users sharing this service.

**Figure 2:**
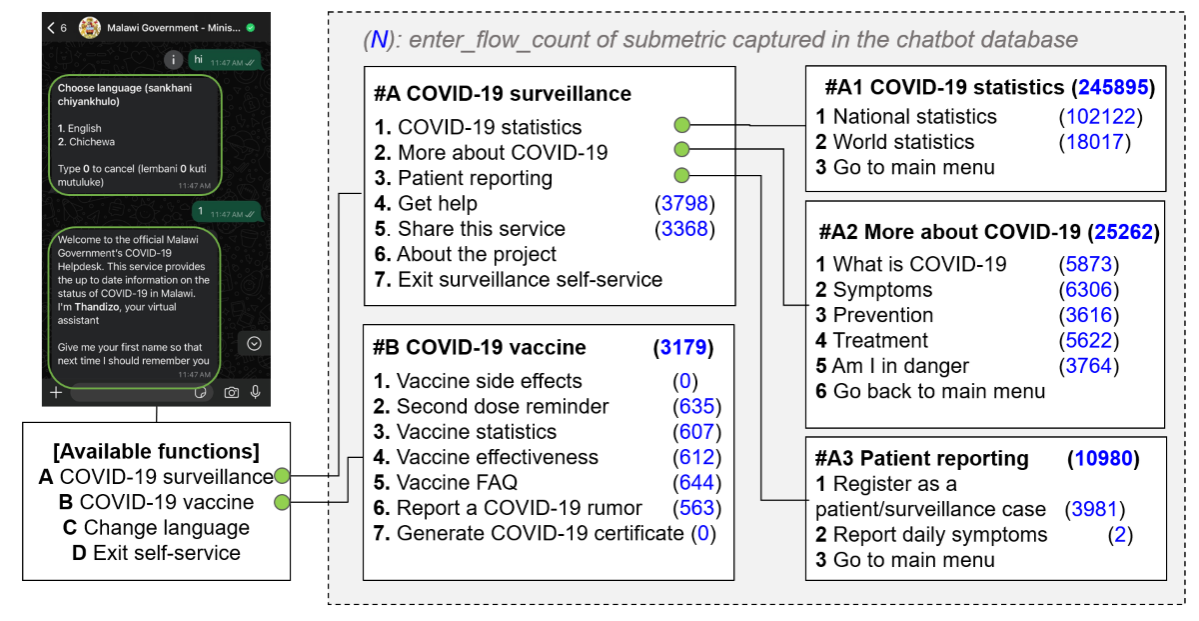
WhatsApp chatbot chat flow. FAQ: frequently asked questions.

Figure 2 shows the chat flow and available functions within the WhatsApp chatbot, which operates through a text-based platform offering predetermined choices and response options. The chatbot offers English- and Chichewa-language options. Approximately 10.9% (37,794/347,117) of accesses involved use of the language change feature, although the selected language was not recorded. Users could access the chatbot without providing personal identifying information. This design does not allow continuity across sessions, requiring a restart of the dialogue with each use. The distribution of accesses across the chatbot’s primary functions was as follows: 70.8% (245,895/347,117) for COVID-19 statistics, 7.3% (25,262/347,117) for general COVID-19 information, 3.2% (10,980/347,117) for patient reporting, and 0.9% (3179/347,117) for COVID-19 vaccine–related services (Table S1 in Multimedia Appendix 1).

The chatbot included functions related to COVID-19 information, transmission, and vaccination. In the “More about COVID-19” section, the most frequently accessed topics were symptoms (6306/25,262, 25%) and COVID-19 definitions (5873/25,262, 23%). Other topics within this section included treatment options (5622/25,262, 22%), danger signs (3764/25,262, 15%), and prevention measures (3616/25,262, 14%). The “COVID-19 Statistics” section accounted for a large proportion of accesses (245,895/347,117, 70.8%), providing users with worldwide and national case statistics. The chatbot also included COVID-19 vaccine–related information, such as side effects, vaccine efficacy, and frequently asked questions.

**Figure 3:**
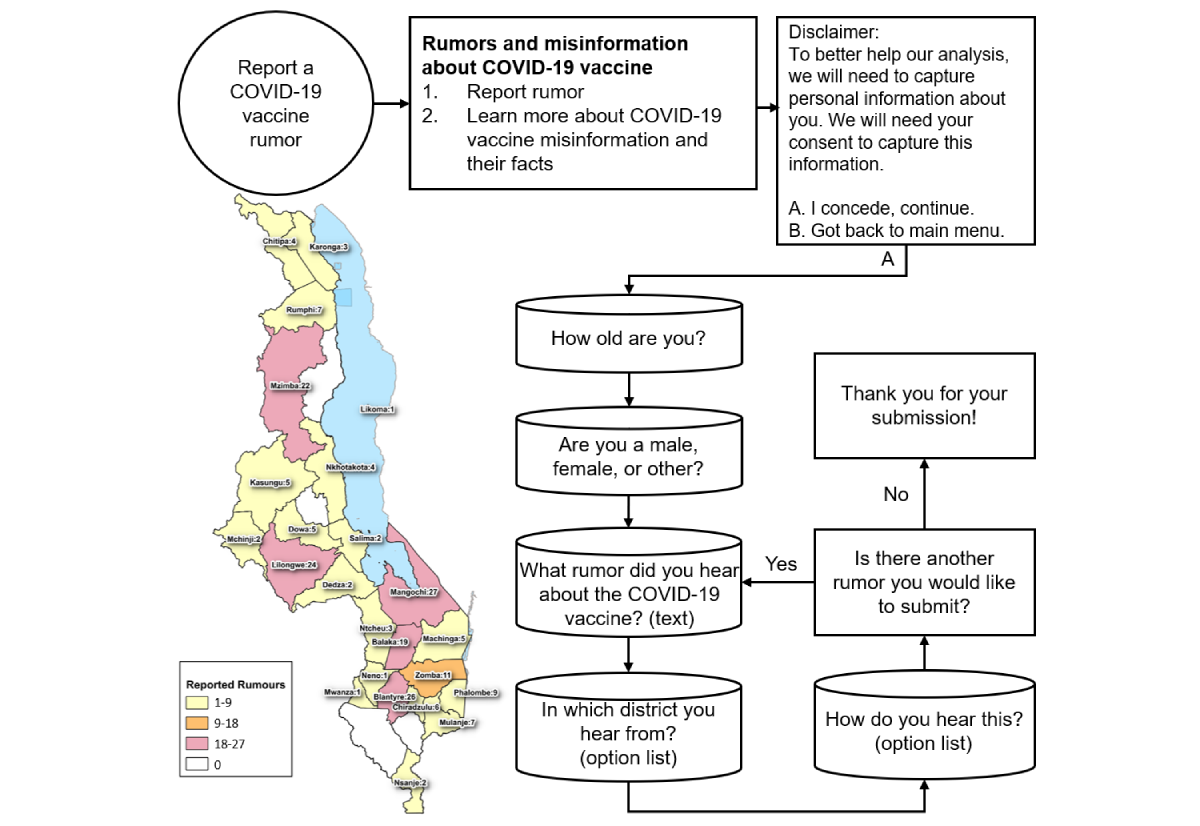
Rumor reporting workflow and geographical distribution of reported rumors.

The “Patient Reporting” service allowed users to self-register suspected COVID-19 cases and report their signs and symptoms. This service accounted for 3.2% (10,980/347,117) of accesses, with 36% (3,981/10,980) resulting in a completed self-registration process. The chatbot also included a vaccine rumor reporting feature to capture and address misinformation related to COVID-19 vaccines. The dialogue data collected through this function included information on characteristics of reported rumors (eg, type, source, and geographical distribution) as well as selected demographic details of reporters (Figure 3). Of the 563 accesses to the rumor reporting feature, 198 (35.2%) resulted in fully submitted rumors, of which 147 (74.2%) were categorized as negative and related to vaccine side effects (Table 1). Reported sources of these rumors included friends or family (42/198, 21%), the media (57/198, 29%), and overheard conversations (66/198, 33%).

**Table 1:** Characteristics of the rumors reported through the WhatsApp chatbot (n=198).

Type of rumor	Source, n (%)					
	Friend or family (n=42)	Health worker (n=17)	Media (radio or WhatsApp; n=57)	Overheard conversation (n=66)	Other (n=16)	Total by type
Negative	34 (81.0)	8 (47.1)	45 (78.9)	53 (80.3)	7 (43.8)	147 (74.2)
Positive	3 (7.1)	2 (11.8)	2 (3.5)	4 (6.1)	0 (0)	11 (5.6)
About COVID-19	3 (7.1)	2 (11.8)	3 (5.3)	1 (1.5)	3 (18.8)	12 (6.1)
Nonsensical	2 (4.8)	5 (29.4)	5 (8.8)	2 (3.0)	4 (25)	18 (9.1)
Questional	0 (0)	0 (0)	2 (3.5)	6 (9.1)	2 (12.5)	10 (5.1)

The geographical distribution of reported rumors (Figure 3) showed a higher number of reports from Mzimba (22/198, 11.1%), Lilongwe (24/198, 12.1%), Mangochi (27/198, 13.6%), Balaka (19/198, 9.6%), and Blantyre (26/198, 13.1%), accounting for 59.6% (118/198) of all negative rumors. These figures represent the absolute number of reports, not incidence proportions as denominator data were not available.

In the context of the “COVID-19 Vaccine” service, the most frequently accessed features were the second dose reminder (635/3179, 19.9%) and frequently asked questions (644/3179, 20.3%), each accounting for 20% of the total accesses. The workflow for managing second dose vaccination reminders involved an integrated process with the One Health Surveillance Platform (OHSP) vaccine registry. This process included verification of the user’s mobile number within the OHSP vaccine registry, with the option to input an Expanded Programme on Immunization number if the mobile number was not found. The chatbot then queried the OHSP vaccine registry using an identifier to retrieve vaccination data, including the scheduled date for the second dose. This integration enabled the retrieval of vaccination records and the delivery of reminder messages to users.

**Figure 4:**
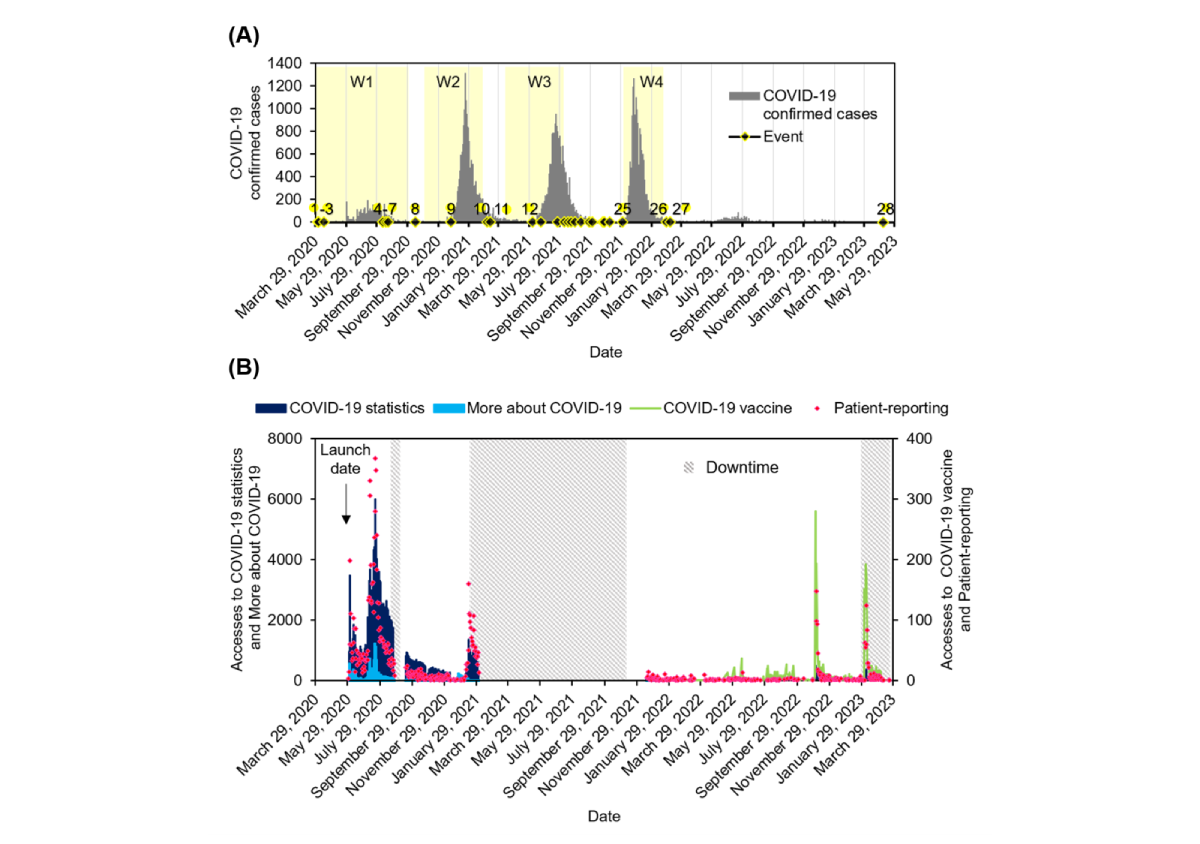
Time trends of (A) new COVID-19 confirmed cases and (B) WhatsApp chatbot use for each main function. The waves (W) of COVID-19 were defined with reference to Anscombe et al [16]. W1: April 2020 to October 2020; W2: November 2020 to March 2021; W3: April 2021 to August 2021; W4: December 2021 to March 2022.

Figure 4 [16] shows the temporal trends of COVID-19 cases alongside chatbot use disaggregated by the primary functions, and Table 2 summarizes COVID-19–relevant official events. Malawi recorded its first COVID-19 case on April 2, 2020 (event 1), followed by the announcement of a national lockdown on April 14, 2020 (event 3). Following the first wave of the outbreak and implementation of social closure measures, the WhatsApp chatbot recorded its highest peak in accesses on July 19, 2020, with 9493 total accesses. Most accesses were related to COVID-19 statistics (n=6012, 63.3%), knowledge of COVID-19 (n=1232, 13%), and self-reporting (n=367, 3.9%). A subsequent decline in both reported COVID-19 cases and chatbot accesses was observed. In August 2020, the Malawi High Court overruled the lockdown policy, and schools were reopened (events 5 and 6). During the second wave, there was a resurgence in chatbot accesses, particularly for COVID-19 statistics and self-reporting. Peak levels of both reported COVID-19 cases and chatbot use were observed between December 2020 and January 2021, a period during which Malawi closed its borders due to a surge in imported cases of COVID-19 (event 9). Following this period, the WhatsApp chatbot experienced a long period of downtime, during which recorded accesses declined until the launch of the COVID-19 vaccine–related features in December 2021.

The chatbot was inaccessible for 38% (407/1070 days) of the study period. These downtimes occurred during 3 periods: August 24, 2020, to September 15, 2020; February 1, 2021, to December 16, 2021; and March 23, 2023, to May 3, 2023. The initial downtime coincided with the early development phase of the system. The longest period of downtime occurred in 2021 and overlapped with the period of COVID-19 vaccine rollout and the introduction of vaccine-related functionalities. Following the introduction of vaccine-related services, 2 spikes in accesses were observed between November 3 and 8, 2022, and February 2 and 8, 2023. These increases were primarily associated with access to vaccine-related features, including rumor reporting and frequently asked questions.

Overall, WhatsApp chatbot use showed temporal alignment with reported COVID-19 cases, particularly during the first and second waves of the pandemic. Increases in use were also observed during periods of social restrictions. Periods of system downtime coincided with reduced recorded use, suggesting the influence of system availability on engagement. The observed increase in use following the introduction of vaccine-related services suggests that updates aligned with emerging public health needs may be associated with renewed user engagement. These observations highlight considerations for the design and maintenance of digital health tools, including system reliability and responsiveness to evolving information needs.

During the COVID-19 pandemic, the EOC call center and the chatbot were used to disseminate information and provide support. The EOC call center operated at the national level and formed part of the country’s risk communication and surveillance system. Due to the increased demand, the EOC call center’s services were extended to CCPF. Since 2009, CCPF has been providing free, community-based health information and support, offering a decentralized system for addressing individual public health inquiries.

**Table 2:** COVID-19–relevant event list.

Event number	Date	Event description
1	April 2, 2020	Malawi registered its first case of COVID-19
2	April 3, 2020	PHIM^a^ EOC^b^ was activated
3	April 14, 2020	Announcement of national lockdown due to COVID-19
4	August 9, 2020	New COVID-19 prevention measures were published in the Government Gazette
5	August 10, 2020	The lockdown policy was overruled by the Malawi High Court
6	August 15, 2020	Announcement of the planning of schools reopening
7	August 20, 2020	Guidance on home-based management of persons with asymptomatic and mild COVID-19
8	October 13, 2020	Reopening of schools for all students
9	December 23, 2020	Malawi closed borders due to surge in imported cases of COVID-19
10	March 5, 2021	MOH^c^ receives the first doses of the COVID-19 vaccine
11	March 11, 2021	Rollout of the first dose of the COVID-19 vaccine
12	June 4, 2021	Rollout of second doses of the COVID-19 vaccine
13	June 21, 2021	Rollout of digital verification of COVID-19 certificate
14-18, 20-23, 26, and 27	July 2021-August 2021, September 2021-October 2021, and February 2022-March 2022	Vaccines were received in Malawi
19	September 8, 2021	eVax certificate for COVID-19 was introduced
24	November 5, 2021	The COVID-19 Vaccination Express Program was launched
25	November 30, 2021	The government introduced additional measures for containing the COVID-19 spread
28	May 5, 2023	The WHO^d^ declared the end of COVID-19 as a public health emergency

^a^PHIM: Public Health Institute of Malawi.

^b^EOC: emergency operation center.

^c^MOH: Ministry of Health.

^d^WHO: World Health Organization.

**Figure 5:**
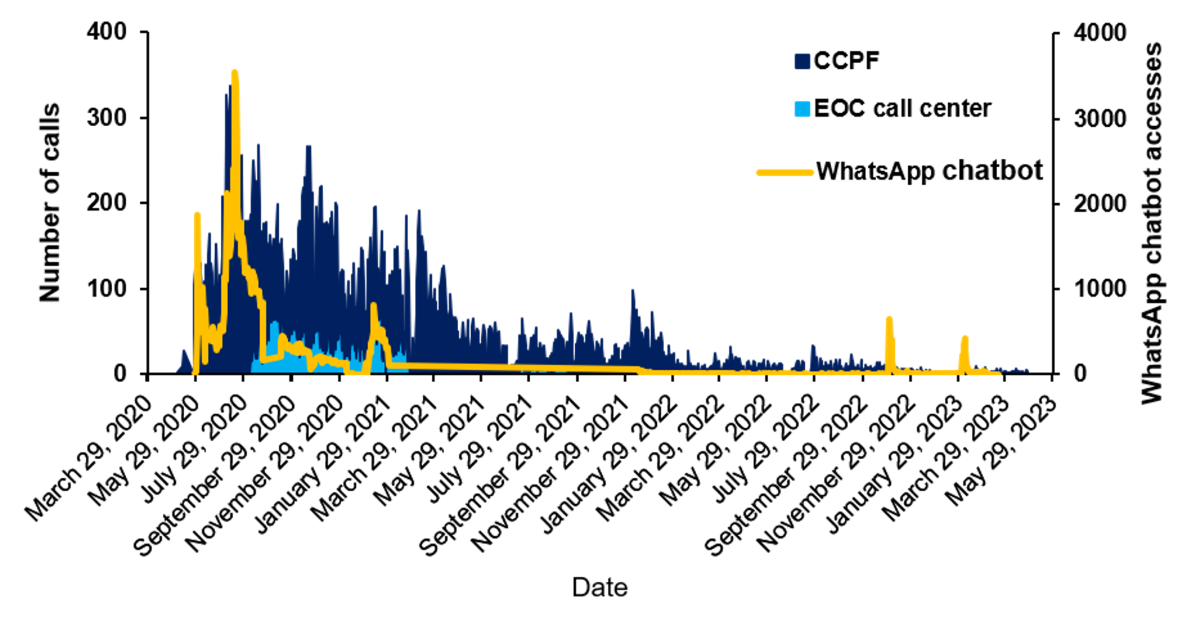
Comparison of COVID-19 information access routes: call center vs WhatsApp chatbot (Chipatala cha pa Foni [CCPF] data collection transitioned to an electronic system on May 6, 2020; emergency operation center [EOC] call center operations were suspended from February 27, 2021, to July 3, 2021, due to unpaid bills).

Figure 5 compares use trends across the 3 information access routes. During the pandemic, 6736 people accessed the EOC call center, and 79,947 accessed CCPF. Of these 86,683 total accesses, 44,214 (51%) were related to COVID-19–specific inquiries. The WhatsApp chatbot recorded 347,117 user interactions during the same period. In January 2020, Malawi had approximately 8.58 million mobile connections, of which 2.81 million (32.8%) were internet users and 510,000 (5.9%) were social media users [18]. These figures provide context for the potential user base of each platform.

However, CCPF received 2196 calls from users reporting symptoms such as fever, cough, and body weakness compared to just 2 symptom reports registered via the WhatsApp chatbot. These use patterns reflect variation in the types of interactions recorded across the platforms.

## Discussion

This study describes the national deployment, functionality, and use patterns of the Malawi MOH COVID-19 WhatsApp chatbot from May 2020 to May 2023. The chatbot (launched on May 29, 2020) recorded 347,117 user accesses during the COVID-19 pandemic. Use patterns broadly aligned with COVID-19 case trends, particularly during the first and second pandemic waves. Information seeking, predominantly access to COVID-19 statistics, accounted for most of the interactions, whereas more complex functions such as self-reporting and rumor reporting were used less frequently. System downtime, most notably during the vaccine rollout period, substantially reduced recorded engagement, whereas the subsequent introduction of vaccine-related services was associated with renewed use spikes. The predominance of information-seeking behavior observed in this study is consistent with other research on digital health tools and chatbots during the COVID-19 pandemic, which were primarily used to provide standardized on-demand health information at scale [1,5,9,12]. COVID-19 statistics alone accounted for 70.8% (245,895/347,117) of all accesses, reflecting a strong public demand for real-time epidemiological updates during periods of uncertainty. This pattern mirrors broader trends observed across Africa, where social media platforms and chatbot-based tools were widely adopted for health communication [4,11]. The lower engagement with the self-reporting and rumor reporting functions aligns with evidence suggesting that users are less inclined to engage with more complex, sensitive, or personally demanding interactions through automated platforms [5,11]. The higher volume of symptom reporting recorded through call-based services such as CCPF compared to just 2 symptom reports via the chatbot further supports this, suggesting that users may prefer human-mediated channels for more personal or sensitive health disclosures. This highlights an important distinction between chatbots as information dissemination tools and call centers as platforms for more personalized support. The temporal alignment between chatbot use and COVID-19 case trends, particularly the peaks observed during the first and second waves and during periods of social restrictions such as lockdowns and border closures, suggests that public engagement was driven by perceived risk and information demand. The increased use during periods of social restrictions may reflect greater reliance on remote information sources. This finding is consistent with research on health-seeking behavior during infectious disease outbreaks, where uncertainty and fear tend to increase reliance on accessible digital information sources [3,5]. The chatbot’s integration with platforms such as OHSP for vaccine scheduling highlights the potential of digital tools to be woven into the fabric of existing digital health systems. The surge in use following the introduction of vaccine-related services suggests that timely updates aligned with evolving public health needs can reinvigorate chatbot engagement [10,11]. Conversely, the 38% (407/1070 days) system downtime, most prolonged during the critical national vaccine rollout period in 2021, likely undermined public trust and weakened user connection with the platform. During inactive periods, users may have turned to less reliable information sources, potentially increasing exposure to misinformation. This underscores the importance of robust infrastructure and contingency planning to ensure system reliability as a foundational requirement for any digital public health tool.

The integration of the chatbot with existing digital health infrastructure such as the OHSP vaccine registry for second dose reminders demonstrates the potential for chatbots to function as an embedded component of broader health systems rather than stand-alone tools. The chatbot acted as a “middle layer” [19], bridging the health sector and the public by offering scalable, consistent, and standardized communication during a fragile period. Offering multiple complementary information access routes such as the chatbot, the EOC call center, and CCPF enabled a diversified communication strategy that combined broad digital outreach with more responsive, personalized support, catering to varied user needs and digital literacy levels.

This study has several limitations. The use of aggregated interaction data did not allow for the identification of unique users, which may overestimate actual reach. The chatbot recorded only the number of accesses to each function without user demographic information; therefore, the characteristics of chatbot users and potential patterns of user behavior could not be adequately examined. It is important to note that the descriptive design does not support causal inference and that the periods of system downtime may have influenced observed use patterns. In addition, variations in access to mobile devices and internet connectivity and digital literacy across Malawi and the sub-Saharan region [18-20] were not directly assessed, which may have had a bearing on the overall reach.

The findings of this study carry broader implications for the design, implementation, and sustainability of digital health communication tools in low- and middle-income countries. The Malawi experience demonstrates that existing platforms such as WhatsApp can be effectively leveraged to support national public health responses even in resource-constrained settings and in ways that may outpace adoption in higher-income countries. Digital tools can offer important strategic support to mainstream health services, extending reach, speeding up communication, and improving public access to health information. However, sustaining engagement requires more than initial deployment. It demands continuous content relevance, robust technical infrastructure, and strategic integration within the wider health system. As artificial intelligence–powered features become increasingly accessible, their incorporation into chatbot platforms could enhance personalization and responsiveness provided that strict safeguards are in place to ensure accuracy and prevent misinformation. Future research should prioritize evaluating user experience, health outcomes, and equity of access to build a stronger evidence base for chatbot-based public health interventions in Africa and similar settings.

## Appendix

Multimedia Appendix 1 Access to specific functions within the whatsapp chatbot.

## Abbreviations

CCPF: Chipatala cha pa Foni
EOC: emergency operation center
MOH: Ministry of Health
OHSP: One Health Surveillance Platform
WHO: World Health Organization

## Data Availability

The datasets generated or analyzed during this study are not publicly available due to government restrictions and ethics approval conditions. However, they are available from the corresponding author on reasonable request and subject to permissions from the Ministry of Health.
