# Supplementary material for: Describing a National Chatbot Deployed by the Ministry of Health in Malawi During the COVID-19 Pandemic: Retrospective Data Analysis
Source: J Med Internet Res. 2026 Jul 16;28:e80960. doi: 10.2196/80960 (PMC13424752; doi:10.2196/80960)
Supplement: Multimedia Appendix 1 [file jmir_v28i1e80960_app1.docx]

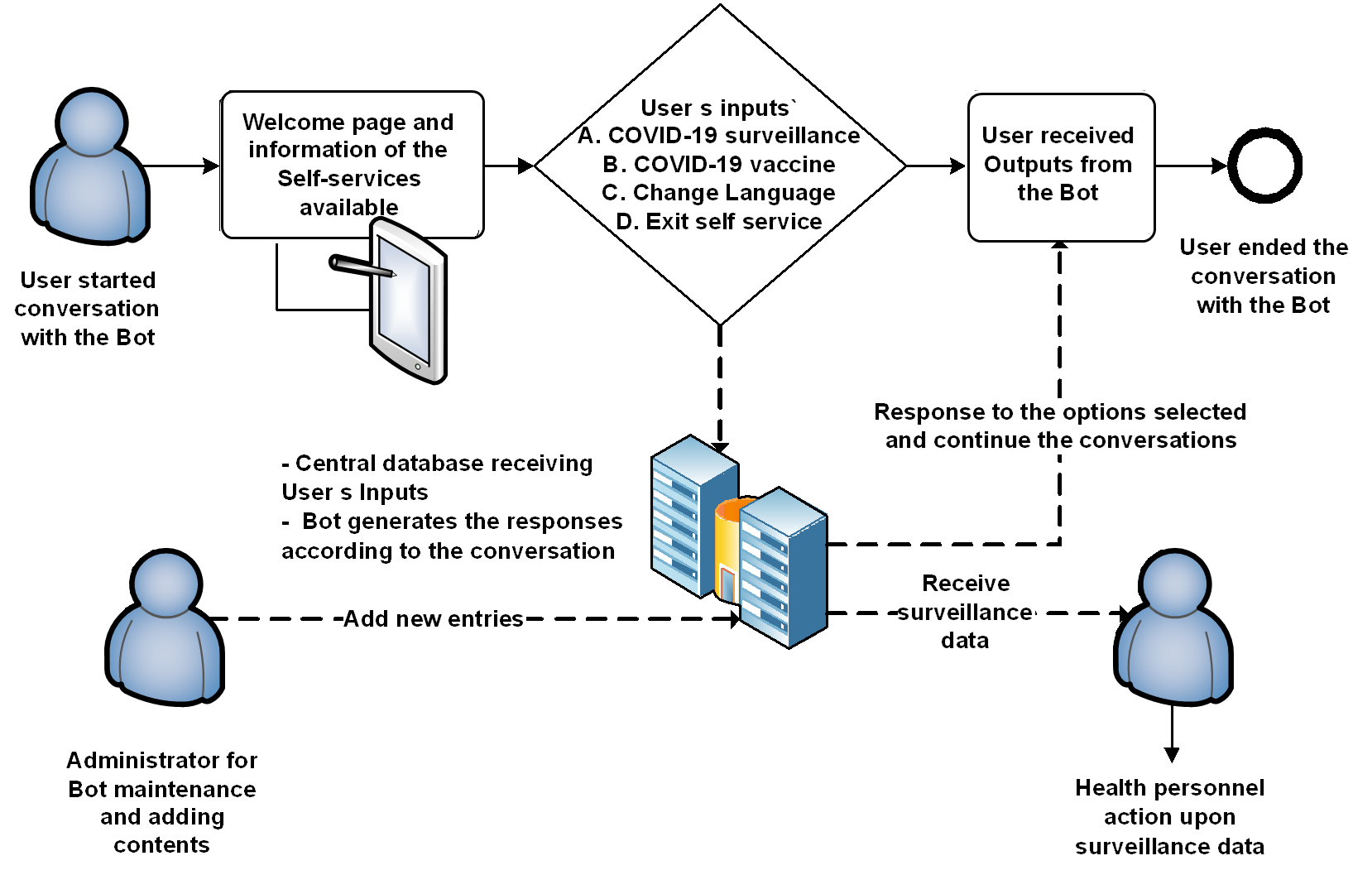


**Figure S1** Malawi COVID-19 ChatBot operation conceptual process flow

**Table S1** Access to specific functions within the Whatsapp chatbot

| **Function** | **Accesses** | **Percentage** |
| --- | --- | --- |
| **A1. COVID-19 Statistics** | **245,895** | **-** |
| A1.1 National Statistics | 102,122 | 41.5% |
| A1.2 World statistics | 18,017 | 7.3% |
| **A2. More about COVID-19** | **25,262** | **-** |
| A2.1 What is COVID-19 | 5,873 | 23.2% |
| A2.2 Symptoms | 6,306 | 25.0% |
| A2.3 Prevention | 3,616 | 14.3% |
| A2.4 Treatment | 5,622 | 14.9% |
| A2.5 Am I in danger | 3,764 | 14.9% |
| **A3. Patient Reporting** | **10,980** | **-** |
| A3.1 Registry as a patient | 3,981 | 36.26% |
| A3.2 Report daily symptoms | 2 | 0.02% |
| **B. COVID-19 Vaccine** | **3,179** | **-** |
| B.1 Vaccine Side Effects | 0 | 0.0% |
| B.2 Second Dose Reminder | 635 | 20.0% |
| B.3 Vaccine Statistics | 607 | 19.1% |
| B.4 Vaccine Effectiveness | 612 | 19.3% |
| B.5 Vaccine FAQ | 644 | 20.3% |
| B.6 Report a COVID-19 Rumour | 563 | 17.7% |
| B.7 Generate COVID-19 Certificate | 0 | 0.0% |
| (Vaccine detail) | 118 | 3.7% |

Note: The COVID-19 vaccine function was launched in January 2020
